# Homobrassinolide Delays Huanglongbing Progression in Newly Planted Citrus (*Citrus sinensis*) Trees

**DOI:** 10.3390/plants13091229

**Published:** 2024-04-29

**Authors:** Meritxell Pérez-Hedo, Alberto Urbaneja, Fernando Alférez

**Affiliations:** 1 Instituto Valenciano de Investigaciones Agrarias (IVIA), Centro de Protección Vegetal y Biotecnología, CV-315, Km 10.7, Moncada, 46113 Valencia, Spain; meritxell_p@hotmail.com (M.P.-H.); urbaneja_alb@gva.es (A.U.); 2 Horticultural Sciences Department, Southwest Florida Research and Education Center, University of Florida/IFAS, Immokalee, FL 34142, USA

**Keywords:** brassinosteroids, citrus greening, *Diaphorina citri*, immune response, infection rates, plant health

## Abstract

Huanglongbing (HLB), or citrus greening, is a devastating disease impacting citrus trees worldwide, with severe effects particularly noted in Florida. Current strategies to combat HLB focus on aggressive replanting, despite the high susceptibility of young trees to infection. In this context, it is critical to explore agronomic practices that can enhance the health and resistance of young citrus trees to HLB. Here, we demonstrate that treatment with homobrassinolide (HBr), a type of brassinosteroid, in newly planted citrus (*Citrus sinensis*) trees can delay HLB infection and improve tree health amidst the high psyllid pressure conditions endemic to Florida. Our study reveals a significant reduction in HLB infection rates in HBr-treated trees compared to control trees, with only 25% of treated trees testing positive for HLB by six months, in contrast to 100% infection in untreated trees. This delay in infection may be attributed to HBr inducing an immune response and negatively impacting psyllid performance, as subsequently demonstrated in a greenhouse experiment. Our findings suggest that HBr applications could serve as a viable strategy to enhance the resilience of citrus production against HLB, underscoring the need for further investigation into their mechanisms of action and potential role in a comprehensive pest and disease management strategy.

## 1. Introduction

Huanglongbing (HLB), also known as citrus greening, is the main threat facing citrus worldwide [1,2]. In Florida, the disease is associated with the Gram-negative α-proteobacterium *Candidatus* Liberibacter *asiaticus* (CLas), with the Asian citrus psyllid (ACP) *Diaphorina citri* Kuwayama (Hemiptera: Liviidae) being the natural vector [3,4,5]. Currently, HLB is considered endemic in Florida [6]. Since the disease was first detected in 2005, the citrus acreage and fruit production have been reduced by more than 70% in Florida [6,7] and continue to decline. The bacteria propagate within the phloem of the citrus tree, producing blotchy mottle and chlorotic patterns on the leaves, callose deposition in the phloem, canopy dieback, root loss, poor-quality fruit, and premature fruit dropping [8,9,10].

The decline in mature trees over the years due to the disease has driven the industry to adopt a very aggressive replanting strategy for new trees, either in whole blocks after removing dead trees or by replacing individual affected trees (resetting). The risk of new plantings becoming infected is exceptionally high because young trees frequently flush, attracting more psyllids. These psyllids feed on the new flushes and transmit the bacteria to the plant’s vascular system, making them more vulnerable to infection than mature trees [11,12]. This is particularly important in the case of reset trees, as the surrounding environment usually contains mature declining trees that are heavily infected, which act as a source of inoculum for the psyllids. In this scenario of widespread infection and severe symptoms of HLB in Florida, agronomic practices that improve plant health in the presence of the disease are needed. 

It has been shown that epibrassinolide, a form of brassinosteroid (Br), could reduce the bacterial titers and alleviate the symptoms of greening in HLB-affected Mexican lime (*Citrus aurantifolia*) and Valencia sweet orange (*Citrus sinensis*) trees in trials in Cuba. The effect seems to be mediated by the activation of many defense-related pathways in the tree [13]. Brs are a unique class of polyhydroxylated steroidal phytohormones. Among the more than 70 types of Br reported so far, 24-epibrassinolide and 28-homobrassinolide are the two most active forms, which are also available commercially [14]. Br signaling was initially studied in the context of growth and development. Still, more recently, the functional roles of Br in stress responses have been accumulating [15,16,17], and several crosstalk models of Br signaling and immune signaling have been proposed. Brs apparently integrate immune system function with normal growth and developmental programs, thus serving as essential regulators of the innate trade-off between disease resistance and plant growth. This seems to be plant-species-specific, with disparate effects [18]. The role of Brs in plant responses to pathogens also appears to be complex, as the regulation of immunity occurs at multiple levels [19,20,21]. Recently, it was demonstrated that Brs could coordinate and enhance the salicylic acid-mediated immune responses in *Arabidopsis thaliana* [22]. Interestingly, the interplay between jasmonic acid (JA) and Brs in modulating the responses to phloem-feeding insects has also been recently elucidated in plants. For instance, Br induced JA in response to phloem-feeding planthoppers in rice (*Oryza sativa*) [23]; moreover, in citrus, Canales et al. [13] showed that allene oxidase synthase, a key enzyme in the JA synthesis pathway, was induced by epibrassinolide treatment.

In Florida, a formulation of homobrassinolide (HBr) is available and labeled for use in citrus at the commercial level. Studies on the effects of HBr on responses to plant disease or biotic stress are still scarce [19,20,21,22,23], and, to the best of our knowledge, such studies have not yet been conducted in citrus (*Citrus sinensis*). The aim of this work was to evaluate the effect of HBr in delaying the development of HLB in *C. sinensis* trees in an area where HLB is widespread. Our results provide the first evidence that HBr, when applied to newly planted citrus (*Citrus sinensis*) trees in an area of high psyllid pressure and endemic HLB conditions, can reduce the rate of HLB infection and improve tree performance. The possible mechanisms of action are presented and discussed.

## 2. Results

### 2.1. Field Experiment

**Figure 1:**
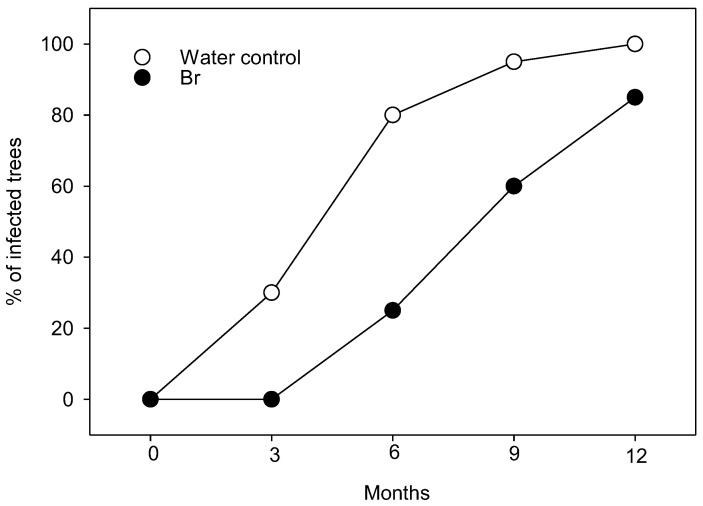
Percentage of trees infected by HLB in HBr-treated plants and control plants in the field trial located in the experimental plots of SWFREC.

**Figure 2:**
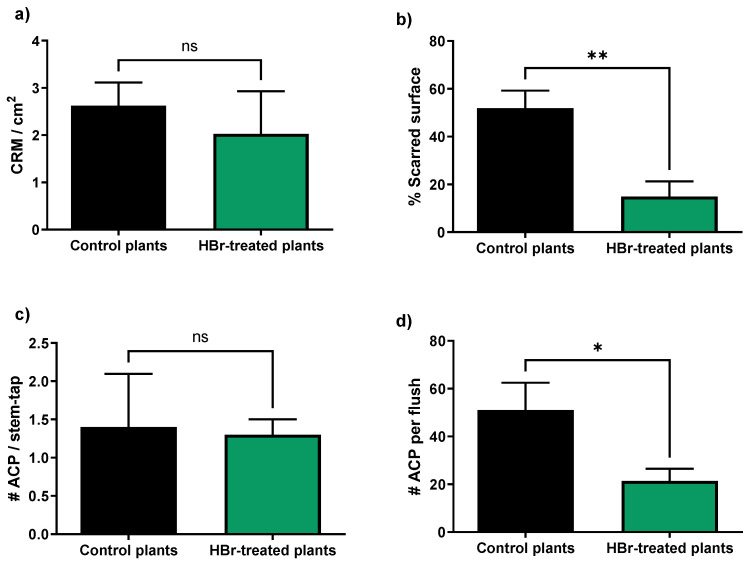
Number (#) (mean ± SE) of citrus rust mites (CRM) per cm^2^ of orange fruit peel surface (**a**); percentage (mean ± SE) of scarred surface area of orange fruit peel (**b**); number (mean ± SE) of adults of *D. citri* (ACP) per stem-tap sampling (**c**); and number (mean ± SE) of eggs, nymphs, and adults of *D. citri* per flush (**d**) for HBr-treated plants and control plants in the field trial located in the experimental plots of SWFREC. * and ** indicate significant differences at the 5% and 1% levels by *t*-test. “ns” indicates no significance by *t*-test.

In this work, we assessed the effect of HBr treatments on HLB incidence in newly planted citrus trees. Trees were planted from the nursery when they were 18 months old. Then, the trees received HBr treatment monthly. After planting, 80% of trees that did not receive the HBr treatment tested positive for HLB by 6 months, and, by 1 year, all trees were infected (Figure 1). In contrast, only 25% of trees treated monthly with HBr tested positive for HLB by 6 months, and some trees (15%) were still HLB-negative after 1 year (Figure 1). After one year post-transplantation, during which the trees had not received any pesticide treatment, it was decided to assess the status of two key pests in Florida citrus cultivation: the citrus rust mite (CRM) *Phyllocoptruta oleivora* (Ashmead) (Acari: Eriophyidae) and the ACP. To do this, the attack of the CRM was evaluated first by counting the number of mites per surface and the percentage of surface scarred by CRM on the fruit peel. The number of CRM was similar in both treatments (*t* = 0.5838; df = 1, 8; *p* = 0.575; Figure 2a), but the scarred surface was significantly smaller in fruits sampled from HBr-treated trees compared to those from the control (*t* = 3.800; df = 1, 8; *p* = 0.0052; Figure 2b). To sample ACP, an initial adult population was estimated using stem-tap sampling, which yielded similar results in both treatments (*t* = 0.138; df = 1, 8; *p* = 0.894; Figure 2c). However, upon further evaluation of the number of eggs, nymphs, and adults present per tender flush, a smaller number of individuals was found in the flushes of HBr-treated trees compared to the control (*t* = 2.358; df = 1, 8; *p* = 0.046; Figure 2d). 

### 2.2. Greenhouse Experiment

**Figure 3:**
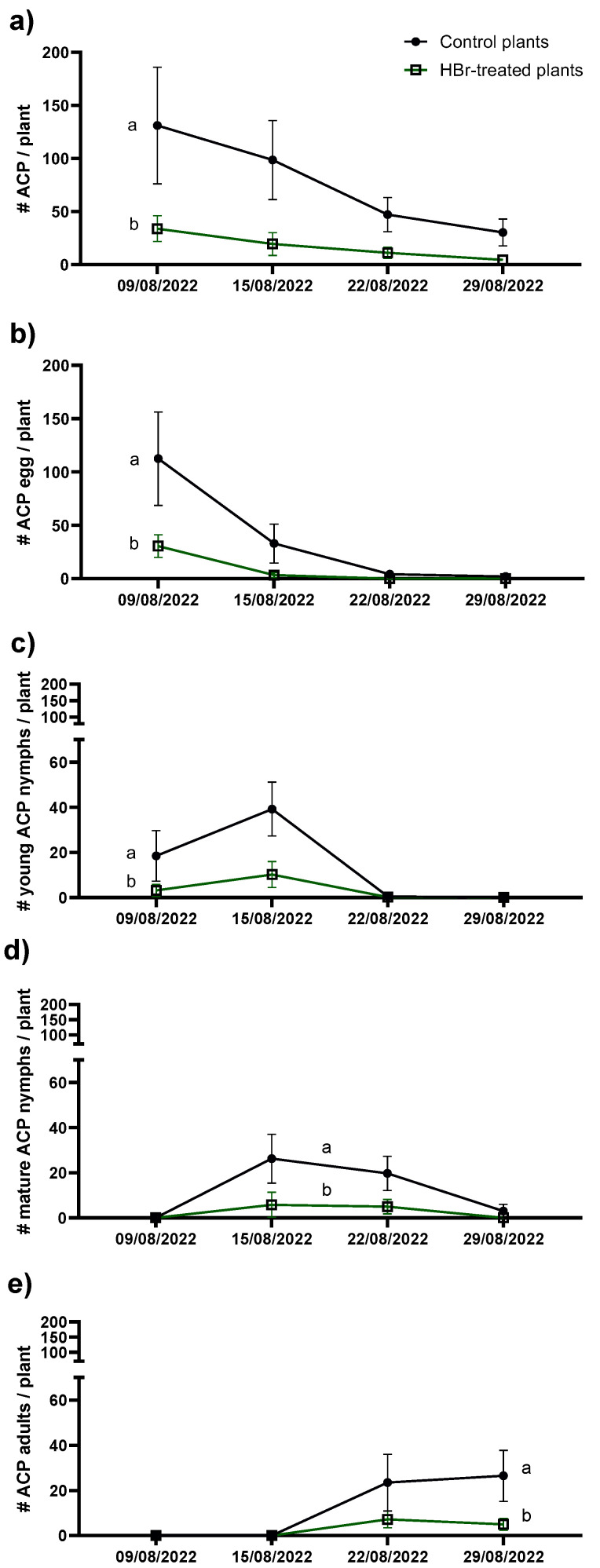
Number (#) of total *D. citri* (ACP) (eggs + nymphs + adults) (**a**), number of eggs (**b**), number of young nymphs (**c**), number of mature nymphs (**d**), and number of adults (**e**) per plant per week (mean ± SE) for HBr-treated plants and control plants. Different letters between treatments indicate significant differences (GLMM, Repeated Measures; *p* < 0.05).

To confirm the results obtained in the field and delve deeper into the effects that HBr treatments may have on ACP, we decided to set up a greenhouse experiment under controlled conditions. To accomplish this, we released five pairs of *D. citri* per two-year-old plant (Valencia grafted onto X-639). We assessed their performance on plants previously treated with HBr, followed by weekly treatments, comparing them with control plants treated with water. The number of *D. citri* individuals that developed on HBr-treated plants was significantly lower than that in the control treatment (*F* = 28.823; df = 1, 34; *p* < 0.001) (Figure 3a). When we analyzed the population dynamics of *D. citri* by developmental instar/stage, these differences persisted across all cases. The number of eggs laid was higher in the control group compared to the HBr-treated plants (*F* = 14.794; df = 1, 34; *p* = 0.001) (Figure 3b). Accordingly, the number of young nymphs (N_1_, N_2_, and N_3_) and the number of mature nymphs (N_4_ and N_5_) was higher in the control treatment than in the HBr-treated plants [*F* = 7.101; df = 1, 16; *p* = 0.012 and *F* = 5.703; df = 1, 25; *p* = 0.025, respectively) (Figure 3c,d). Additionally, the resulting number of adults was also more significant in the control treatment compared to the HBr-treated group (*F* = 9.701; df = 1, 36; *p* = 0.007) (Figure 3e). The structure of the sampled population data allowed us to estimate the mortality rates for each stage transition of *D. citri*, enabling us to observe that the mortality of *D. citri*, including eggs, nymphs, and total (from egg to adult), was higher in the HBr-treated plants than in the control plants (Table 1).

**Figure 4:**
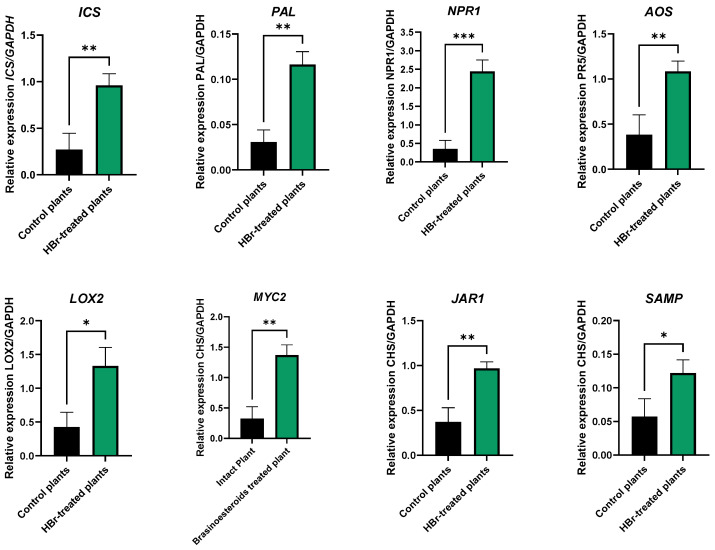
Transcriptional responses of the plant defense marker genes isochoristame synthase (*ICS*; from the upstream SA biosynthetic pathway), phenylalanine ammonia-lyase (*PAL*; from the upstream SA biosynthetic pathway), nonexpressor of pathogenesis-related genes 1 (*NPR1*; from the downstream SA biosynthetic pathway), allene oxide synthase (*AOS*; from the upstream JA biosynthetic pathway), lipoxygenase 2 (*LOX2*; from the upstream JA biosynthetic pathway), transcription factor *MYC2* (*MYC2*; from the downstream JA biosynthetic pathway), jasmonate resistant 1 (*JAR1*; from the downstream JA biosynthetic pathway), and small antimicrobial peptide marker (*SAMP*), in HBr-treated plants and control plants. Data are presented as the mean independent transcript expression value relative to the constitutive *GAPDH* gene ± SE. *, **, and *** indicate significant differences at the 5%, 1%, and 0.01% levels by *t*-test.

In addition to observing the psyllid’s performance, we studied how this treatment affected the plants’ immune systems by analyzing the expression of salicylic acid, jasmonic acid, and antimicrobial peptide (*SAMP*) markers 48 h after the first application of HBr. In response to HBr treatment, the differential regulation of genes was observed in both the salicylic acid and jasmonic acid metabolic pathways, along with the *SAMP* marker, with higher expression in the HBr-treated plants. In the salicylic acid pathway, the upstream genes isochorismate synthase (*ICS*) and phenylalanine ammonia-lyase (*PAL*) showed significant increases in expression (*t* = 3.295, df = 1, 7, *P* = 0.006 and *t* = 4.276, df = 1, 7, *p* = 0.009, respectively), indicating the early activation of this pathway. Simultaneously, there was a notable surge in the expression of the downstream gene nonexpressor of pathogenesis-related genes 1 (*NPR1*) (t = 5.167, df = 1, 7, *p* = 0.001), indicative of the robust activation of the associated defense mechanisms. In the jasmonic acid pathway, the upstream genes allene oxide synthase (*AOS*) and lipoxygenase 2 (*LOX2*) increased in expression (*t* = 3.009, df = 1, 7, *p* = 0.010 and *t* = 2.472, df = 1, 7, *p* = 0.021, respectively), while the downstream genes transcription factor MYC2 (*MYC2*) and jasmonate resistant 1 (*JAR1*) also showed significant increases (*t* = 4.072, df = 1, 7, *p* = 0.024 and *t* = 2.917, df = 1, 7, *p* = 0.011, respectively), indicating pathway activation and the modulation of the plant’s response. Lastly, the *SAMP* peptide marker showed an increase in expression (*t* = 2.014; df = 1, 7; *p* = 0.042) induced by HBr treatment (Figure 4).

## 3. Discussion

Our results provide evidence that treatment with homobrassinolides (HBr) has an impact in terms of reducing the incidence of Huanglongbing (HLB) in newly planted citrus under high psyllid pressure and endemic HLB conditions in Florida. This study adds to the growing body of knowledge about the positive physiological effects of brassinosteroids, such as the advancement of flowering, the acceleration of fruit maturation, and increased fruit yields [24,25,26]. Furthermore, our work aligns with previous studies that demonstrated the capacity of other brassinosteroids to enhance the health of citrus affected by HLB through the activation of plant defense pathways [13]. 

In our greenhouse experiment, HBr treatment induced the differential regulation of genes in the SA and JA metabolic pathways and increased the expression of an antimicrobial peptide marker (*SAMP*). The coordination between the salicylic and jasmonic acid pathways and the induction of *SAMP* illustrates a sophisticated mechanism by which HBr may confer citrus plants with an increased capacity to resist or delay HLB progression.

It has been suggested that Brs enhance SA-mediated defense responses, and it has been proposed that an adequately sized endogenous Brs pool should be maintained to support SA responses and downstream signaling [22]. Our data strongly support this finding, as the exogenous Brs treatment of young citrus plants induced both pathways leading to SA biosynthesis, the chorismate mutase and isochorismate synthase pathways, and also induced downstream immune-related gene expression.

The early activation of the salicylic acid pathways, as indicated by a significant increase in the expression of the *ICS* and *PAL* genes, suggests that HBr treatment could prime plants for a rapid and effective response against pathogens. This early activation is crucial, as salicylic acid plays a central role in acquired systemic resistance [27], allowing the plant to bolster its defenses before the pathogen can establish itself. Furthermore, the notable increase in *NPR1* expression, a key regulator of salicylic acid-mediated gene expression, underscores the potent activation of related defense mechanisms, potentially explaining the delay in HLB progression in HBr-treated plants. *NPR1* is crucial in controlling HLB due to its role in regulating the immune balance of plants [28,29,30,31]. CLas infection triggers unbalanced immune responses, leading to the overaccumulation of callose and reactive oxygen species (ROS), which causes phloem obstruction and the development of HLB symptoms [32]. However, it has been discovered that the overexpression of the *AtNPR1* gene from *A. thaliana* in susceptible varieties confers robust HLB tolerance [33]. The overexpression of *AtNPR1* suppresses the overaccumulation of callose and ROS induced by CLas in citrus and *Arabidopsis*, respectively [34]. The function of *NPR1* is centered on its interaction with SA, where *NPR1* acts as an SA receptor, promoting redox changes that convert *NPR1* from an oligomeric complex to monomers that move to the nucleus. There, *NPR1* interacts with TGA transcription factors to activate the transcription of defense genes, including *PR* genes [34]. Sarkar et al. [34] suggest that *NPR1* overexpression is a promising route for the development of HLB-tolerant citrus through genetic manipulation. In our work, we have demonstrated that plants treated with HBr significantly overexpress the *NPR1* gene.

Concurrently, the activation of the jasmonic acid pathway, evidenced by the increased expression of the *AOS* and *LOX2* genes, along with the significant increases in *MYC2* and *JAR1*, reflects the modulation of another critical pathway in the plant’s response to stress and pathogen attacks. This pathway, complementary to the salicylic acid pathway, is associated with defense against herbivores and certain types of microbial infections [35,36], suggesting an integrated defense strategy that could limit the effectiveness of HLB vectors, as observed in the greenhouse experiment. The *AOS* and *LOX2* genes play essential roles in the initial stages of JA biosynthesis. At the same time, *MYC2* and *JAR1* are fundamental in the signaling and response to JA, modulating the expression of defensive genes and resistance to herbivores [35]. The observed overexpression of *AOS* and *LOX2* indicates the activation of JA metabolism in response to HBr treatment, suggesting increased JA production. *MYC2*, a key transcription factor in the JA pathway, and *JAR1*, involved in the conjugation of JA with isoleucine to form the active JA–Ile complex, also showed increased expression. This confirms the pathway’s activation and suggests the refined modulation of the plant’s defensive response upon HBr treatment. This activation of the JA pathway could explain the relationship between HBr treatment and increased *D. citri* mortality in HBr-treated plants. The overexpression of these genes indicates that plants can potentially amplify their arsenal of defensive responses, which includes the production of toxic or repellent secondary metabolites for herbivores, the induction of defense-related proteins such as protease inhibitors, and the fortification of the physical structures of the plant [37]. The results obtained in the greenhouse experiment can explain the observed decrease in the populations of *D. citri* and the damage caused by the CRM on HBr-treated plants in the field experiment. Previous research demonstrates the connection between JA signaling and herbivore resistance, showing that increased protease activity induced by JA can reduce the incidence of mites and other herbivores on plants [38,39,40,41,42,43]. These proteases interfere with protein digestion in herbivores, limiting their growth and survival in the host plant [44,45]. Therefore, HBr treatment appears to exert a systemic effect on plants, inducing JA-mediated defense responses resulting in reduced susceptibility to herbivore attacks. Although the quantity of CRM was similar in both treatments, the surface affected by these mites on the fruit was significantly smaller in HBr-treated plants, suggesting that while the treatment did not directly affect the mite population, it did improve the plant’s ability to mitigate the damage caused by them. This protective effect could be related to the induction of specific proteins or changes in the plant surface composition that hinder mite feeding or reproduction. The lower quantity of ACP on the tender shoots of HBr-treated plants also highlights the treatment’s impact in reducing herbivore viability.

The increase in SAMP peptide expression underscores a broad response to stress induced by HBr treatment, suggesting that HBr activates specific defense pathways and enhances the plant’s ability to handle stress, potentially improving its overall resilience to various threats. This peptide was described by Huang et al. [46] with potent antimicrobial activity that directly targets CLas but also activates innate immunity in citrus trees, offering both therapeutic and preventative capabilities. In our case, the HBr treatment increased its content in the HBr-treated plants. 

The finding that HBr delays HLB progression in young trees opens up an exciting possibility in areas where HLB is endemic, such as Florida. Currently, citrus growers in Florida are increasingly using psyllid exclusion methods, such as individual protective covers (IPCs). These are polyethylene mesh bags with pores smaller than the size of the psyllid’s body, effectively excluding the insect vector. The effectiveness of this tool in maintaining newly planted citrus trees free from disease has been recently reported [47,48]. IPCs have proven to be a promising strategy to protect young trees from HLB, keeping them symptom-free and negative for HLB in trials. Trees under IPCs show faster growth and higher chlorophyll accumulation, suggesting that this approach could be helpful in extending tree productivity and improving the health of infected ones. However, eventually, IPCs have to be removed due to tree growth (typically 2 or 3 years after planting), leaving young trees exposed to infection. Any treatment that could prolong tree health is highly desirable in this scenario. We are currently investigating the effectiveness of a system that combines a physical barrier (IPCs) followed by treatment with HBr.

In conclusion, HBr treatment represents a promising strategy for HLB management in citrus, offering an additional tool in the arsenal against this disease. The role of brassinosteroids, specifically HBr, in modulating the plant immune response to biotic stress is becoming increasingly evident. However, much remains to be elucidated. Future research should focus on understanding more thoroughly the underlying molecular mechanisms of HBr’s observed effects and optimizing the dosage and application timings to maximize the benefits under commercial conditions. Additionally, it would be interesting to explore the interaction between HBr and other HLB management methods, such as the aforementioned IPCs, the integrated management of ACP and other citrus pests, and their effect on different citrus varieties, to develop more holistic and sustainable management approaches.

## 4. Materials and Methods

### 4.1. Field Experiment 

#### 4.1.1. Plant Material, Experimental Design, and Treatments

Six replicated plots of three 18-month-old ‘Valencia’ (*Citrus sinensis*) trees on ‘Cleopatra’ (*Citrus reshni* hort. ex Tanaka) rootstock per treatment (6 replicas × 3 trees × 2 treatments = 36 trees total) in a randomized complete block design were planted at the Southwest Florida Research and Education Center in Collier County, Florida (26.466966 N, 81.446917 W). Trees were fertilized using conventional granular fertilizer (8N-4P-8K; Diamond R, Fort Pierce, FL, USA). Irrigation was by under-tree microjets. No insect management was performed in the experimental block during the experiment. Treatments were water (control) and 1 μM HBr prepared in water (homobrassinolide 0.1%, Repar Corp, Silver Spring, MD, USA) and were performed monthly for 1 year.

#### 4.1.2. Candidatus Liberibacter Asiaticus (CLas) Detection

For bacterial infection estimation, leaves were collected from each individual tree every 3 months. Detection was performed as in [48]. Briefly, three to four mature leaves from recent flushes were randomly collected from the middle tree of each replicated plot every 3 months for 1 year. The petioles and midribs of leaves were excised, minced with a razor blade, lyophilized in a FreeZone 6 freeze–dry system (Labconco, Kansas City, MO, USA), and pulverized using a mini bead beater (Biospec products, Inc., Bartlesville, OK, USA). According to the manufacturer’s instructions, DNA from 100 mg of pulverized leaves was extracted using the Wizard Magnetic 96 DNA Plant System (Promega Corporation, Madison, WI, USA). DNA was quantified using a microplate reader (Synergy HTX Multimode Reader, Biotek Instruments, Inc., Winooski, VT, USA) and normalized to 10 ng/µL. CLas was detected by quantitative real-time polymerase chain reaction (qRT-PCR) using a 7500 Fast Real-Time PCR system (Applied Biosystems, Foster City, CA, USA). The primers and probes used for detection were HLBas and HLBr and probe HLBp, respectively, as described in [49]. Samples with a Ct value of less than 36 were considered CLas-positive. 

#### 4.1.3. CRM and ACP Samplings 

The trees set fruit for the first time 1 year after planting. We estimated the damage caused by CRM and their number per fruit. For this purpose, we counted, in 8 randomly selected fruits on each central tree of each repetition, the number of mites present in 1 cm^2^ of the fruit surface using a 15× magnifying lens (SPI supplies, West Chester, PA, USA). Two measurements were taken per fruit. Additionally, the scarred surface caused by CRM damage was estimated for each fruit [50]. In each of the trees, the population of adult *D. citri* present was estimated using two stem-tap samples [51], and, from two randomly selected shoots with tender leaves, the number of eggs, nymphs, and adult *D. citri* present was counted.

### 4.2. Greenhouse Experiment 

#### 4.2.1. Plants and Insects

Two-year-old “Valencia” cultivar plants, grafted onto rootstock “X-639,” a hybrid cross of Cleopatra mandarin (*Citrus reshni* (Tanaka)) and *Poncirus trifoliata* (L) Raf., were used. These citrus plants were obtained directly from a certified nursery (Southern Citrus Nurseries, Dundee, FL, USA). They were left undisturbed for two months, without receiving any chemical treatment, in an isolated and well-protected greenhouse before being used in the experiments. Three weeks before the start of the experiments, the plants were defoliated entirely and pruned, leaving 4–5 lateral branches on each one, with approximately 4–5 buds. They were allowed to re-sprout in the greenhouse where the experiment was conducted, each inside its corresponding cage. After three weeks, all the plants had an average of feather shoots of 7.2 ± 0.8, the preferred state for ACP oviposition [52].

Adults of *D. citri* were obtained from colonies established at the Southwest Florida Research & Education Center (SWFREC). These colonies were reared on *Murraya paniculata* (L.) Jack plants, with initial specimens collected from experimental fields at SWFREC. A binocular stereo microscope (SPI Supplies, West Chester, PA, USA) was used to isolate the psyllids and distinguish between females and males.

#### 4.2.2. Diaphorina Citri Performance on HBr-Treated Plants

The experiment was conducted in a 20 × 6 m greenhouse located at SWFREC. The greenhouse was accessed through a double door. One datalogger (HOBO U23 Pro v2 External Temperature/Relative Humidity Data Logger, HOBO Dataloggers, Bourne, MA, USA) was placed in the center of the greenhouse to record the temperature and relative humidity. This experiment was conducted in August 2022 and the environmental conditions include an average night/day temperature in the greenhouse of 34/20 °C and the relative humidity varied between 45% and 96%. Natural photoperiods were about 11 h. 

Five replicates were conducted for each treatment to be tested. The two treatments included water (control) and 1 μM HBr, which were directly sprayed onto the plants 48 h before releasing the *D. citri* adults and then weekly. Each replicate involved introducing a plant with receptive flushes for *D. citri* oviposition into an individual entomological cage measuring 60 × 60 × 90 cm (Insect Rearing Cage, Entomological Livestock Supplier, Parma, Italy), with five pairs of *D. citri* carefully selected and placed on the plants. Plants were watered twice a week. Four weekly evaluations assessed the numbers of *D. citri* (adults, eggs, and nymphs) per plant.

#### 4.2.3. RNA Extraction and qRT PCR Analysis of Gene Expression

For gene expression determination, the apical parts from five control and five treated plants were collected 48 h after HBr treatment. Each sample was crushed in liquid nitrogen using a mortar and pestle. Total RNA from 0.1 g of fresh leaf tissue was extracted with the RNeasy Plant Mini Kit (Qiagen, Valencia, CA, USA), following the manufacturer’s instructions. The RNA was quantified, and its purity was confirmed using a Nanodrop. Following the manufacturer’s instructions, 1 µg of total RNA was used for gDNA removal and cDNA synthesis using the QuantiTect Reverse Transcription Kit (Qiagen, Valencia, CA, USA). The cDNA samples were stored at −80 °C to further assay the defense genes’ expression in citrus.

Real-time PCR amplification was performed in a 7500 Fast Real-Time PCR System (Applied Biosystems, Foster City, CA, USA), using the NZYSupreme qPCR Green Master Mix (2×) (NZYTech, Lisboa, Portugal). Reactions were performed in a 10 μL volume containing 0.5 μM of each primer and 1 μg of cDNA template. The cycling program was set to 2 min of the pre-cycling stage (95 °C), 40 cycles of 5 s at 95 °C, 25 s at 58 °C, and 15 s at 72 °C. The primer sequences of defensive genes ICS, PAL, NPR1, AOS, LOX2, MYC2, JAR1, and SAMP and the housekeeping gene *GADPH*, used as a standard control gene for normalization, are represented in Table 2.

### 4.3. Statistical Analysis 

A generalized linear mixed model (GLMM) was employed to analyze the total number of ACP, eggs, and young and mature instars, allowing for repeated measures. We utilized a Poisson distribution and a logarithmic link function to detect differences between the HBr-treated plants and the control group. The treatment was considered a fixed factor, while the week was treated as a random one. To explore variations among treatment levels, a post hoc analysis was conducted using the LSD test (*p* < 0.05). Data with zero values (such as nymphs in the first week) were excluded from the statistical analysis. The mortality rates in both treatments were compared using a *t*-test (*p* < 0.05).

## Figures and Tables

**Table 1:** Mortality rates of *D. citri* at different developmental stages on control and HBr-treated plants, along with corresponding statistical analysis (*t*-test; *p* < 0.05).

Mortality	Control Plants	HBr-Treated Plants	Statistics
Egg	49.9 ± 16.3	65.0 ± 10.2	*t*_1,7_ = 0.817; *p* = 0.441
Nymphal	35.9 ± 18.2	68.6 ± 11.2	*t*_1,7_ = 1.600; *p* = 0.158
Egg–adult	76.6 ± 5.33	85.7 ± 6.8	*t*_1,7_ = 1.008; *p* = 0.347

**Table 2:** List of genes and primers used to analyze the expression of defense-associated genes in citrus.

Gene	Gene Name	Citrus ID	Primer Sequence (5’→3’)
*GAPDH*	*Glyceraldehyde-3-phosphate dehydrogenase*	LOC102624117	FW: GGAAGGTCAAGATCGGAATCAA
			RV: CGTCCCTCTGCAAGATGACTCT
*PAL*	*Phenylalanine ammonia-lyase-like*	LOC102620464	FW: CACATTCTTGGTAGCGCTTTG
			RV: AGCTACTTGGCTGACAGTATTC
*ICS*	*Isochorismate synthase 2, chloroplastic*	LOC102630235	FW: GGAGGAGGAGAGAGTGAATTTG
			RV: GGGTTGCTTCCTTCTACTATCC
*NPR1*	*BTB/POZ domain and ankyrin repeat-containing protein*	LOC102617188	FW: GTACCTTGAAAACAGAGTTGGACTGG
			RV: TGCTCCTCTTGCATTTTGAAAGGTG
*MYC2*	*Transcription factor MYC2*	LOC102626457	FW: TGCATCTACAGCCGACCC
			RV: TAGGTCCAGCCCTCACGA
*LOX2*	*Linoleate 13S-lipoxygenase 2-1, chloroplastic-like*	LOC102629656	FW: GAACCATATTGCCACTTTCG
			RV: CGTCATCAATGACTTGACCA
*AOS*	*Allene oxide synthase*	AY243478	FW: AGATCTTATTCCCGAACATGGT
			RV: CGGACTTCATCAACGGCAT
*JAR1*	*Jasmonate resistant 1*	LOC102611440	FW: AAGGCGATGCAGTCACAATG
			RV: TGGTGGAAATCAGGACCAAAG
*SAMP*	*Response A/B barrel domain-containing protein HS1*	LOC102628374	FW: AACAGGGGCAAGAATGTGAGCAT
			RV: ACACGTACTGTTGTCGGTTTGTAGTCA

## Data Availability

The original contributions presented in the study are included in the article; further inquiries can be directed to the corresponding author.
